# Defining the competency of professionalism at two military medical centers

**DOI:** 10.5116/ijme.59c6.2634

**Published:** 2017-10-09

**Authors:** Mary Edwards, Virginia Randall, Clfton Yu, Gary Crouch, Chris Foster

**Affiliations:** 1 Department of Surgery, San Antonio Military Medical Center, San Antonio, USA; 2 Department of Pediatrics, Uniformed Services University of the Health Sciences, Bethesda, USA

**Keywords:** Professionalism, military medical centers, clinical competencies, Uniformed Services University of the Health Sciences, United States Department of Defense

## Introduction

The subject of professionalism has received much attention in recent literature, and in the United States is listed in both the Liaison Committee on Medical Education (LCME) standards^1^^-^^4^ and the Accreditation Council on Graduate Medical Education (ACGME)^5^ clinical competencies as an essential component of pre-and postgraduate medical education. While definitions and assessment methods for professionalism abound, what is lacking is an understanding of how learners understand and define professionalism. Adult learning theory posits that adults learn best when the material being taught relates to their learning situation and helps them approach problems they are encountering in real life.^6^ Through better appreciation of the learner’s perspective and understanding of professionalism, educators can develop more efficient and targeted curricula. We surveyed resident physicians at two large military training centers to understand their perspective on professionalism.

### Our teaching environment

San Antonio Military Medical Center (SAMMC) and Walter Reed National Military Medical Center (WRNMMC) are the two largest graduate medical education (GME) training platforms in the United States Department of Defense. Between the two centers, a complete spectrum of surgical and medical training programs exist, offering residency and fellowship positions to over 1100 military trainees. Both hospitals are designated teaching hospitals for the Uniformed Services University of the Health Sciences (USUHS) F. Edward Hebert School of Medicine. This medical school exclusively trains physicians for service on active duty in the United States Army, Navy, Air Force and Public Health Service. The University begins professionalism training early in medical school, with small group discussions and dedicated didactics.  Medical students in the second through fourth years spend the vast majority of their educational experience at military hospitals, including SAMMC and WRNNMC, and almost all will continue with residency training in military medical training centers.  Similarly, large proportions (20%) of residents at these two institutions matriculate from USUHS. Therefore, it is important to understand perspectives on professionalism from this spectrum of learners who are fully immersed in the military culture. 

Defining professionalism from the learner’s viewpoint

Following Institutional Review Board approval, we emailed an anonymous survey to all residents and fellows at our two training centers asking the learners to free-text the three essential characteristics of a professional. Approximately one-third of the trainees responded.  The characteristics of patient-centered and life-long learning were by far the most frequently cited as a defining feature of professionalism. Over half of the respondents from any year group incorporated one or both of those themes.  These results were similar to 4th-year medical students in a similar survey of USUHS medical students, but dissimilar to 1st through 3rd-year medical students.^7^ This suggests that patient-centered care and life-long learning may be learned as a desired attribute, or a threshold concept, grasped by most either late in medical school or early in residency.   Further analysis of responses also found that the concepts of professionalism did not vary by specialty, with both medical and surgical trainees articulating the paramount importance of patient-centered care and life-long learning in defining professional behavior in physicians.^8^

### Professionalism as a concept evolves overt raining

Some educators suggest that professionalism, at its core, cannot be taught, implying if a fundamental understanding is not present at the beginning of training, it will never be fully embraced.  This is somewhat supported by data which reveals many residents are facing professionalism lapses in training also suffered similar lapses in medical school, and are at risk to face further negative professional actions following completion of training.^9^  However, our data would suggest that the concepts of professionalism evolve during training and shift to a patient-centered focus late in medical school when clinical rotations begin and when patient-centered care is modeled by senior residents and faculty. Life-long learning is also seen as a focus of these learners and may reflect this generation’s tendency to be more questioning of dogma handed down from an older generation.^10^

### Military impact

It is likely that the military environment in which they train impacted the residents’ perspective. The military in and of itself is an organization predicated on public service; therefore the concept of patient-centered care--placing the patient’s needs and desires first--is very much in keeping with military culture.  Life-long learning is also clearly part of this culture, as military advancement is contingent on obtaining the professional education at all levels of one’s career, as well as fulfilling a broad spectrum of clinical, academic, leadership and administrative roles.  Physicians who do not embrace this continuum of change, challenge and education are not promoted and do not advance.

## Conclusions

We found that trainees in two large US military teaching hospitals largely define the concept of professionalism as patient-centered care and lifelong learning. These characteristics begin to come into focus for 4th-year medical students and are important to residents in every year and every specialty. They are not necessarily innate in early learners but are acquired late in medical school and likely impacted by observing behaviors in senior residents and faculty.  The military environment likely influences this perspective of professionalism, and understanding this point of view is necessary for faculty training these residents.  As faculty, if we fully embrace and demonstrate these concepts to our residents, we will increase our impact and credibility as educators in any teaching or learning circumstance.  By framing resident education through the context of these two elements, we can improve understanding and resident buy-in of professionalism, the critically important, but often undefined aspect of the complete physician. 

### Declaration

The opinions expressed herein are those of the authors and do not represent the official position of the San Antonio Military Medical Center, The Uniformed Services University of the Health Sciences, the United States Army, The United States Navy, the United States Air Force or the United States Department of Defense.

### Conflict of Interest

The authors declare that they have no conflict of interest.
